# Pericentriolar material structure and dynamics

**DOI:** 10.1098/rstb.2013.0459

**Published:** 2014-09-05

**Authors:** Jeffrey B. Woodruff, Oliver Wueseke, Anthony A. Hyman

**Affiliations:** Max Planck Institute of Molecular Cell Biology and Genetics, Pfotenhauerstrasse 108, Dresden 01307, Germany

**Keywords:** pericentriolar material, centrosome, organelle scaling, microtubule-organizing centre

## Abstract

A centrosome consists of two barrel-shaped centrioles embedded in a matrix of proteins known as the pericentriolar material (PCM). The PCM serves as a platform for protein complexes that regulate organelle trafficking, protein degradation and spindle assembly. Perhaps most important for cell division, the PCM concentrates tubulin and serves as the primary organizing centre for microtubules in metazoan somatic cells. Thus, similar to other well-described organelles, such as the nucleus and mitochondria, the cell has compartmentalized a multitude of vital biochemical reactions in the PCM. However, unlike these other organelles, the PCM is not membrane bound, but rather a dynamic collection of protein complexes and nucleic acids that constitute the organelle's interior and determine its boundary. How is the complex biochemical machinery necessary for the myriad centrosome functions concentrated and maintained in the PCM? Recent advances in proteomics and RNAi screening have unveiled most of the key PCM components and hinted at their molecular interactions ( table 1). Now we must understand how the interactions between these molecules contribute to the mesoscale organization and the assembly of the centrosome. Among outstanding questions are the intrinsic mechanisms that determine PCM shape and size, and how it functions as a biochemical reaction hub.

## 1. Pericentriolar material structure

**Figure 1.**
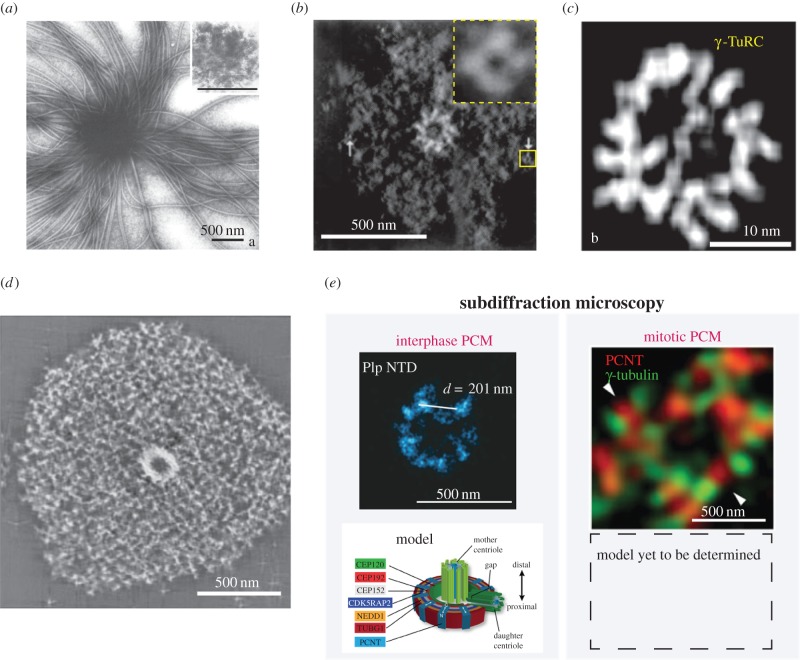
Structural organization of the PCM. (*a*) Negative stain electron micrograph of a centrosome isolated from Chinese hamster ovary cells. One hundred and twenty-five MTs emanate from the densely staining centre. (Adapted from [2].) (*b*) Structure of a purified *Drosophila* centrosome as revealed by electron tomography. A ninefold radially symmetric centriole can be seen at the centre surrounded by PCM. The inset shows a magnified view of a ring-like complex found within the PCM. These complexes measured 25–30 nm in diameter and were determined to contain γ-tubulin. (Adapted from [3].) (*c*) The γ-TuRC was later isolated from *Drosophila* cells and analysed by electron tomography. (Adapted from [4].) (*d*) Harsh treatment of *Spisula* centrosomes with potassium iodide revealed 12–15 nm wide filaments running throughout the PCM, leading to the hypothesis that a lattice-like network forms the structural foundation of the PCM. (Adapted from [5].) (*e*) The development of subdiffraction microscopy techniques allowed high precision localization of proteins within the PCM. The first picture depicts the localization of PCNT-like protein (D-PLP) in *Drosophila* cells determined with stochastic optical reconstruction microscopy (STORM). Comparison of the localization of numerous proteins revealed that the interphase PCM contains ordered subdomains of defined size. However, the expansive PCM that exists during mitosis is less ordered. Localization of PCNT and γ-tubulin in human cells using three-dimensional SIM is shown. (Adapted from [6–8].)

Decades of research have pursued atomic-level resolution of the underlying pericentriolar material (PCM) structure with little avail. This is probably owing to limitations in methodology, but also to the fact that the PCM does not behave like most ordered proteinacious assemblies. In the earliest electron micrographs depicting centrosomes *in situ*, the PCM appeared as a densely stained amorphous mass that surrounded the highly structured centrioles [1]. Electron microscopy (EM) micrographs of centrosomes isolated from mammalian cells did little to resolve the amorphous mass any further, although these experiments definitively showed that microtubules (MTs) originate from the PCM (figure 1) [2] and that PCM integrity is sensitive to chelating divalent cations [9].

The resolution required to distinguish subdomains within the PCM would not be achieved until the implementation of EM tomography. Using this approach, in combination with immunolabelling, Moritz *et al.* [3] could discern gamma-tubulin-containing ring structures 25–30 nm in diameter within PCM from isolated *Drosophila melanogaster* centrosomes [3]. Higher resolution structures of immunoprecipitated *Drosophila* γ-tubulin ring complexes (γ-TuRCs) confirmed the 25–30 nm diameter of these rings and showed that they cap the minus ends of PCM-derived MTs [4]. Similar ring structures were observed in centrosomes isolated from surf clam oocytes and, intriguingly, were stripped away after exposure to potassium iodide, leaving behind an underlying skeletonized lattice of 12–15 nm wide filaments [5]. Unlike the untreated centrosomes, the salt-stripped centrosomes could not nucleate MTs. Interestingly, the ring structures reappeared and MT nucleation could be restored if the salt-stripped centrosomes were incubated in oocyte extract. Taken together, the findings from these studies hinted that the PCM comprises a porous structural scaffold onto which γ-tubulin and other soluble components from the cytoplasm are loaded.

Concurrently, scientists sought to determine the identities and biochemical properties of the proteins that construct the PCM scaffold, or the so-called ‘centromatrix’. Researchers took advantage of the curious fact that auto-immune sera taken from scleroderma patients reacted widely with centrosomes and, thus, could be used as a robust label for specific centrosome proteins in western blot and immunofluorescence assays [10]. Use of these sera revealed that the PCM is a dynamic structure and led to the identification and biochemical characterization of PCM proteins [11]. In this manner, one of the first PCM components, pericentrin (PCNT), was identified, cloned and its necessity for spindle organization described [12]. The discovery of additional core PCM components such as Cep192/SPD-2, CDK5RAP2/Cnn, Cep152/Asterless and SPD-5 in various organisms revealed that the only major similarity among PCM organizing proteins was an abundance of coiled-coil domains [13–18]. The coiled-coil motif consists of intertwined α-helices and is known to mediate protein–protein interactions [19]. Thus, it was proposed that these numerous coiled-coil domains could mediate robust inter-molecular interactions to allow formation of the centromatrix [16,20]. Whether these coiled-coil scaffold proteins *per se* are sufficient to assemble the centromatrix, and whether their coiled-coil domains are critical for this assembly process, has yet to be determined.

Analysis of purified centrosomes by mass spectrometry and large-scale RNAi and localization screens in *Caenorhabditis elegans*, *Drosophila* and human cells unveiled a diverse bounty of centrosome proteins [17,21–25] (table 1). Owing to the diversity and tight clustering of PCM proteins at centrosomes, it is of little surprise that electron microscopy so far could not discern structural information about the PCM. Labelling and observing individual components within the PCM promised to circumvent this problem, but the resolution limitations of conventional light microscopy and immunogold-EM only allowed the localization of the components without generating any meaningful structural insights. However, recent advances in light microscopy technology opened new possibilities for mapping PCM architecture. Four independent studies used subdiffraction light microscopy techniques, such as three-dimensional structured illumination (three-dimensional SIM) and stochastic optical reconstruction microscopy (STORM) to identify the substructures within the PCM [6–8,65]. The authors developed antibodies to label distinct epitopes of different PCM proteins and measured their distances from the outer centriole wall. A key finding was that interphase PCM proteins are distributed in concentric toroids of discrete diameter around centrioles. A subset of proteins, human CDK5RAP2, PCNT, CEP152 and the *Drosophila* orthologues PCNT-like protein (D-PLP) and Asterless, were shown to exhibit highly extended conformations spanning approximately 100 nm from the centriole wall to the outer toroidal domains of the interphase PCM. In stark contrast, analysis of the same proteins during metaphase revealed no ordered structures or discrete distributions. Interestingly, the co-localization between labelled PCM protein pairs was minimal, indicating that, despite the lack of apparent organization, PCM proteins still occupy distinct domains in the metaphase centrosome [7]. These findings argue that the PCM is first assembled in interphase as an ordered, compact layer immediately surrounding the centrioles that then serves as a foundation for the expansive formation of PCM towards metaphase.

**Table 1.** Important proteins for PCM assembly and function. (Key PCM proteins and their homologues from humans, flies and nematodes are grouped based on their general role in PCM biogenesis and function. Scaffold proteins are believed to be involved in forming the foundation of the PCM. The effector proteins are more peripheral factors involved in MT organization. SMC_prok_B: chromosome segregation protein SMC, common bacterial type. PACT_coil_coil: PCNT-AKAP-450 domain of centrosomal targeting protein.)

	*Homo sapiens*	*D. melanogaster*	*Caenorhabditis elegans*	domains	PCM-related phenotypes	reference
scaffolds	Cep192	Spd-2	SPD-2	coiled coil, polo-box-binding domain	centriole duplication defect, reduced PCM, PLK-1 targeting to centrosomes lost	[13,26–28]
	Cep152	Asl (Asterless)		coiled coil, SMC_prok_B (PFAM)	centriole duplication defect, reduced PCM	[29,14]
	CPAP	SAS-4	SAS-4	coiled coil, tubulin binding	centriole duplication defect, reduced PCM	[30,31]
	PCNT	D-PLP		coiled coil, centrosome-targeting (PFAM)	reduced PCM	[12,32–34]
	CDK5RAP2	Cnn (centrosomin)		coiled coil, MT association (PFAM)	reduced PCM, centriole-PCM attachment defect	[15,35–37]
	CG-NAP/AKAP450			coiled coil, calmodulin-binding domain	centriole duplication defect	[38,39]
			SPD-5	coiled coil, SMC_prok_B (PFAM)	reduced PCM	[16]
kinases	Plk1 (polo-like kinase 1)	Plk1	PLK-1	kinase, polo-box	reduced PCM, loss of phosphorylation of Cdk5Rap2/CNN, PCNT, and SPD-5	[40–43]
	AURKA (Aurora A kinase)	Aurora A	AIR-1	kinase	centrosome separation defect, loss of effector recruitment (γ-tubulin, D-TACC, MSPS)	[44–46]
phosphatases	PPP2ca	PP2A	LET-92	phosphatase	centriole duplication defect, loss of MT stability via TPX2 and KLP-7, centrosome–nuclei detachment	[47,48]
	PPP2r1a	PP2A-B	SUR-6	regulatory subunit of PP2A	centriole duplication defect	[49]
			RSA-1	regulatory subunit of PP2A	loss of MT stability	[47]
			RSA-2	regulatory subunit of PP2A	loss of MT stability	[47]
	PP4c	PP4	PPH4.1	phosphatase	abberant pericentrin foci, loss of effectors and kinases (α- and γ-tubulin, PLK-1, Aurora A)	[50–53]
effectors	γ-tubulin	γ-tubulin	γ-tubulin	tubulin	impaired spindle assembly, impaired MT nucleation	[3,54–57]
	TACC2	D-Tacc	TAC-1	coiled coil (TACC domain)	loss of effectors (ZYG-9/ZYG-8), loss of MT stability	[58–61]
	CKAP5(chTOG)	Msps	ZYG-9	MT binding, TOG domain	loss of MT stability, loss of centrosome integrity	[61–64]

## 2. Formation of the inner pericentriolar material layer

How is the centriole-proximal ground layer laid? Mennella *et al*. [6] reported that D-PLP is organized with quasi-ninefold symmetry radiating outwards from the centrioles [6]. Furthermore, the outer boundary of the interphase PCM layer approximately matches the length of either D-PLP in fly embryos [6] or PCNT in human cells [7]. One interpretation of these results is that D-PLP and PCNT serve as molecular rulers to set the size of the inner PCM layer. Gopalakrishnan *et al*. [31] presented data indicating that, in centrosome-free *Drosophila* embryo extracts, D-PLP forms complexes with SAS-4, Cnn and γ-tubulin, all proteins discovered to occupy concentric toroidal domains within the interphase PCM [31]. These data suggest that the organization and size of the interphase PCM is established through the deposition of pre-assembled complexes of defined stoichiometry and size. This proposed model is intriguing but requires more rigorous biochemical assessment of reconstituted PCM proteins before becoming canonical. Studies in *C. elegans* indicate that this model may not apply to all systems. In the one-cell embryo, SAS-4-containing centrioles contributed by the sperm are sufficient to organize functional centrosomes even if the maternal pool of SAS-4 has been depleted by RNAi [30]. Thus, it is unlikely that SAS-4 organizes pre-assembled cytoplasmic complexes essential for PCM assembly in worms. Furthermore, mass spectrometry and fluorescence correlation spectroscopy experiments suggest that the core PCM components in *C. elegans*, SPD-2 and SPD-5, do not interact with each other or with SAS-4 in the cytoplasm, but rather exist as mainly monomeric entities prior to incorporation into the PCM (O. Wueseke 2014, unpublished data). These results argue that PCM assembly through the incorporation of large, pre-assembled units is not a universal mechanism found in all centrosome-containing eukaryotes.

## 3. Expansion of the pericentriolar material in mitosis

**Figure 2.**
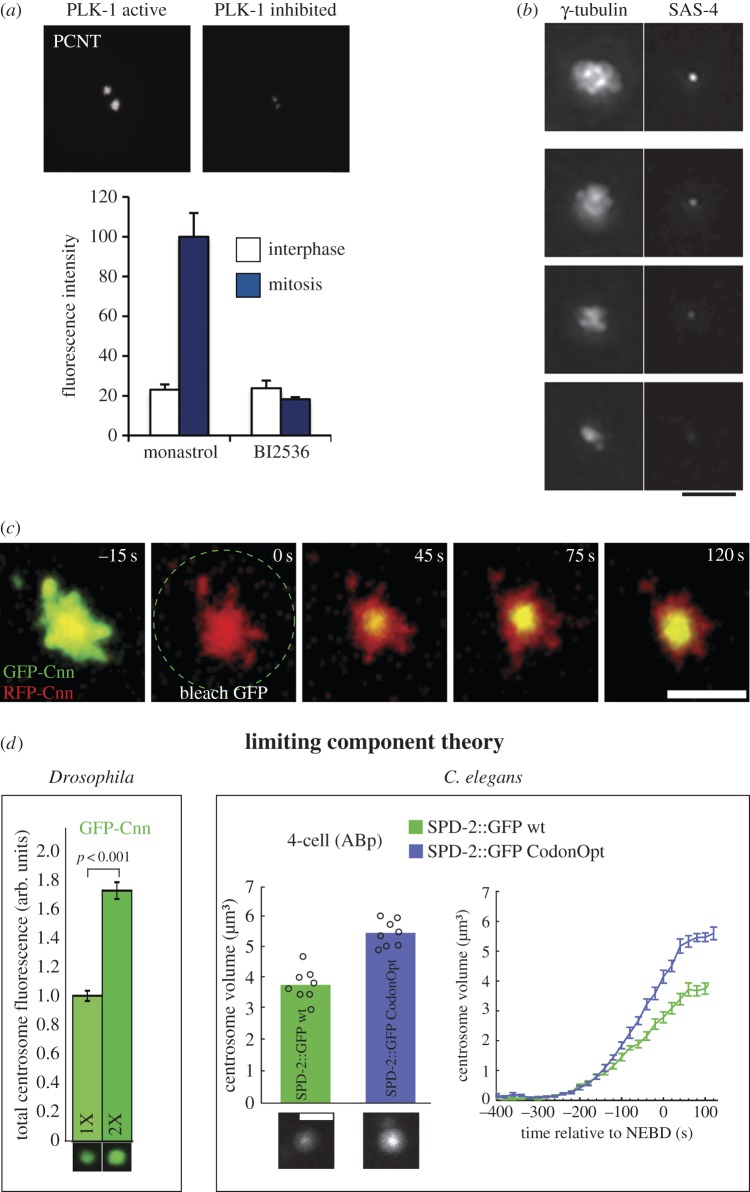
Factors regulating PCM growth and final size. (*a*) Inhibition of PLK-1 kinase activity with the small molecule BI2436 reduces the incorporation of PCNT at centrosomes. Interestingly, inhibition of PLK-1 did not affect PCNT localization to the interphase centrosome. (Adapted from [42].) (*b*) γ-tubulin staining scales proportionally with the amount of SAS-4 localized to centrioles in *C. elegans* embryos, suggesting that centriole duplication and size determine PCM growth. Scale bar, 4 μm. (Adapted from [30].) (*c*) After photo-bleaching, GFP-Cnn first recovers at the centre of the centrosome, near the centrioles and then spreads outward to the periphery in *Drosophila* cells. This result indicates that Cnn is incorporated immediately around centrioles, suggesting that centrioles play a critical role in converting PCM proteins to an assembly-competent state. Scale bar, 3 μm. (Adapted from [35].) (*d*) Overexpression of Cnn and SPD-2 increases the final steady-state size of the PCM in *Drosophila* and *C. elegans*, respectively. SPD-2 expression levels were increased through a codon-optimization strategy for the *C. elegans* experiments (SPD-2::GFP CodonOpt, blue bar and line). These results suggest that Cnn and SPD-2 act as limiting components for the formation of PCM. Scale bar, 3 μm. (Adapted from [35,66].)

How does the expansive outer PCM layer assemble around the established inner PCM? Decades of cell biology and molecular genetics have indicated that this process of expansion is driven, in part, by kinase-regulated incorporation of core scaffolding proteins, MT-associated proteins (MAPs) and MT nucleating complexes. For instance, inhibition of polo kinase prevents accumulation of γ-tubulin at centrosomes and mitotic PCM expansion in humans, flies and worms (figure 2) [23,40,41,67,68]. Furthermore, polo kinase has been demonstrated to directly phosphorylate the core scaffold proteins pericentein, Cnn and SPD-5, and mutation of polo phosphorylation sites in these proteins precludes PCM assembly ([23,69], J. Woodruff and O. Wueseke 2014, unpublished data). Another important role is played by Aurora A kinase, which is necessary for PCM maturation and function by regulating the recruitment of MT-nucleating complexes and MAPs. For example, inhibition of Aurora A prevents recruitment of γ-tubulin and ZYG-9 (chTOG/Msps) in *C. elegans* embryos [44] and Msps and D-TACC in *Drosophila* S2 cells [46]. However, Aurora A inhibition had little effect on SPD-5 localization [16]. These data suggest that PLK-1 drives the assembly of the core structure of the mitotic PCM scaffold, whereas Aurora A stimulates the addition of downstream effector molecules like MAPs to the scaffold.

Interestingly, the requirement for kinase activity to drive PCM expansion can be bypassed in certain non-physiological situations. Overexpression of PCNT or CDK5RAP2 has been shown to artificially promote PCM expansion in interphase-arrested mammalian tissue culture cells. These hypertrophic centrosomes recruit a small amount of γ-tubulin and NEDD1, indicating that they are similar, but not identical, to normal mitotic centrosomes [7,70]. These results hint that the core PCM proteins can self-assemble without external stimulation by mitotic kinases like polo. Under physiological conditions, the effective concentration of the core PCM proteins may be too low to permit spontaneous self-assembly. During mitosis, polo kinase phosphorylation of the core PCM proteins could perhaps stimulate this assembly process. This model is still speculative, but it is consistent with the available data and would allow regulation of PCM assembly in a cell-cycle-dependent fashion.

## 4. Pericentriolar material disassembly

If phosphorylation of key core scaffold proteins drives PCM assembly and maturation, then it is reasonable to assume that dephosphorylation of these targets contributes to PCM dissolution. Indeed, in metaphase-arrested cells with mature centrosomes, acute inhibition of polo kinase activity promotes the removal of PCNT and γ-tubulin from the centrosome in human cells, indicating the existence of a constantly ongoing dephosphorylation reaction [71]. This result suggests that the cell drives the PCM towards assembly or dissolution by modulating the balance of phosphorylation and dephosphorylation activities inside the PCM. Although this hypothesis is tantalizing, a phosphatase that promotes PCM dissolution has yet to be identified. So far, two major phosphatases have been reported to localize to centrosomes and participate in the regulation of PCM dynamics, namely protein phosphatase 4 (PPH4) and protein phosphatase 2A (PP2A) [47,48,50–52,72]. PPH4 was reported to be required for centrosomal recruitment of Aurora A and γ-tubulin, indicating that it plays a role in centrosome maturation [50–52,72]. PP2A, on the other hand, was found to act on centrosomal effector molecules like the MT-destabilizing kinesin KLP-7 as well as TPX-2 to modulate spindle size in *C. elegans* [47]. It will be important to determine in the future whether PP2A, PPH4, or any other phosphatase directly influences PCM dissolution, and or de-phosphorylates PLK-1 substrates needed for PCM assembly.

Cortical forces may also tear apart PCM during mitotic exit. After anaphase completion in *C. elegans* embryos, γ-tubulin::GFP-labelled centrosomes not only become dimmer, indicating the loss of PCM protein, but also rip apart in a cortical-directed manner [66,73]. Elimination of MTs, the dynein heavy chain *dhc1*, or MT attachment sites on the embryo cortex prevent this dramatic rupturing of PCM [73], implicating a MT motor-driven mechanism for PCM dissolution. Although not essential for PCM disassembly, these MT-dependent cortical forces accelerate the process. One potential explanation is that the rupturing of PCM increases the surface to volume ratio of the PCM, thereby expediting the dephosphorylation reaction. A similar mechanism may also exist in flies. PCM fragments often emerge and radiate away from centrosomes in *Drosophila* embryos in a process termed ‘flaring’ [74]. Flaring events were shown to be dependent on MTs and reach maximal intensity during telophase and interphase, coordinate with PCM disassembly. Taken together, these findings indicate that the combination of PLK-1 inactivation, phosphatase activity and MT-dependent forces ensure the rapid disassembly of PCM during mitotic exit.

## 5. Factors regulating pericentriolar material growth kinetics and final size

Like any intracellular organelle, the size of a centrosome is set for any individual cell type. This is especially clear in early *C. elegans* embryos, where the total amount of centrosome material is constant. Altering the number of centrosomes in a one-cell embryo will change the size of each individual centrosome, but the sum of centrosome volume will remain the same. For example, if a one-cell embryo contains four centrosomes instead of two, then each centrosome will be half the expected size [66,75]. What are the factors that determine PCM expansion and its maximum size? Both centriole-dependent and -independent mechanisms seem to be required.

One mechanism centres around the role of centrioles, as centriolar proteins and overall centriole size were found to affect PCM size. Proper centriole duplication is an essential step towards PCM accumulation around centrioles [76]. In *C. elegans* two-cell embryos, partial RNAi-depletion of SAS-4 inhibits centriolar duplication to varying degrees and reveals that PCM size directly correlates with the amount of centriole-localized SAS-4 [30]. This result suggests that centrioles directly determine the incorporation rate of PCM components. This idea is supported by photo-bleaching experiments in *Drosophila* embryos which showed that Cnn incorporates exclusively in the vicinity of centrioles and then migrates outward towards the periphery to form the bulk of the mitotic PCM [35]. Thus, localizing and binding to centrioles may be a universal rate-limiting step for the incorporation of PCM components. Such a mechanism is favourable in that it would also ensure that PCM only forms around centrioles. As SAS-4 is critical for PCM accumulation around centrioles, and that Cnn, D-PLP, γ-tubulin and other PCM proteins have been identified in SAS-4-containing complexes in *Drosophila* [31], SAS-4 could act as a centriolar tether and a key control point for regulating PCM assembly.

There is also evidence that PCM proteins can assemble independently of the centriole, suggesting that an additional process regulates PCM growth kinetics. For example, overexpression of either Cnn or an Asterless mutant that cannot bind Plk-4 induced the formation of ectopic, acentriolar MT organizing centres in *Drosophila* [29,77]. Furthermore, in acentriolar mouse oocytes, PCNT forms perinuclear assemblies that localize γ-tubulin and orchestrate MT behaviour similar to mitotic centriolar-nucleated PCM [78,79]. Whether or not other nucleation sites like the surface of the nucleus mimic the role of the centriole in this case, or whether the process does not require a nucleation site at all, remains to be seen. In total, these results argue that PCM itself can stimulate the formation of additional PCM. Such an autocatalytic mechanism would drastically increase the overall incorporation rate of PCM and is probably essential for the *C. elegans* embryo and other systems that require robust PCM expansion over very short mitotic phases (e.g. in the *C. elegans* one-cell embryo, the PCM enlarges 60-fold in approx. 8 min) [66]. Systems with smaller final centrosome size or extended mitotic phases might not require such a mechanism to accelerate PCM assembly.

Rates for most biological reactions are directly related to the concentration of the starting material, indicating that PCM growth could be set by concentration, and PCM size set by total amount, of PCM proteins within the cytoplasm. This is certainly the case in the *C. elegans* embryo, where centrosome size decreases in direct proportion to the cytoplasmic volume, and thus total available PCM components, during development. Moreover, in this system, overexpression of the limiting component SPD-2 increased the rate of PCM growth and its maximum achievable size [66]. Likewise, in *Drosophila* embryos, increasing the cytoplasmic concentration of Cnn also increased PCM size and growth rate [35]. The fact that recruitment of Cnn to the PCM depends on its interaction with SPD-2 argues that a SPD-2-centred mechanism to regulate PCM growth and size is conserved throughout evolution [35]. Exhaustion of a limiting component could perhaps also explain the characteristic plateau in centrosome size seen during mitosis in both *C. elegans* and *Drosphila* embryos [35,66]. Recent experiments on spindle length in *Xenopus* extracts encased in artificial cells suggest that spindle size is also limited by the availability of certain components like tubulin [80,81]. Thus, a limiting component mechanism could be a general way of setting the size of intracellular organelles in early embryos [75].

## 6. Outlook

### (a) A unified model for pericentriolar material dynamics

**Figure 3.**
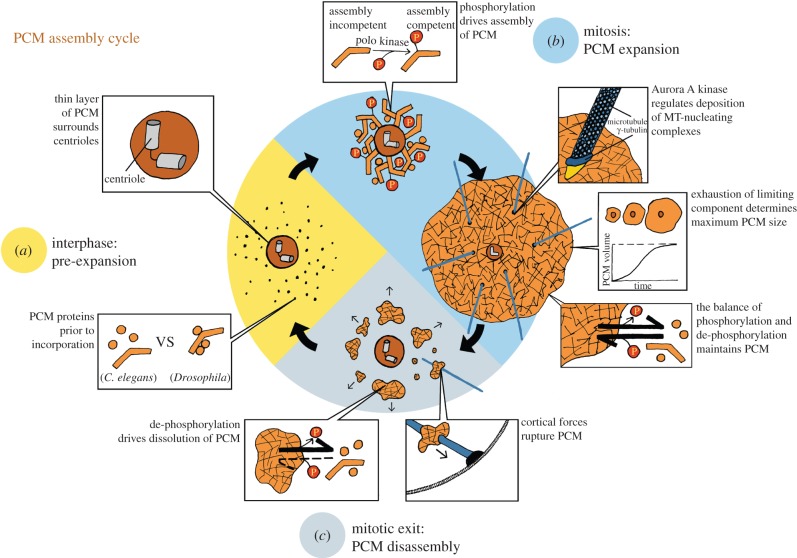
The PCM assembly cycle in embryonic systems. (*a*) During interphase, a thin layer of PCM surrounds the centrioles. In the cytoplasm are unincorporated PCM proteins, and there are species-specific differences in their assembly state. In *C. elegans*, the unincorporated core PCM components are separate entities, whereas in *Drosophila*, the core PCM proteins may already be pre-assembled into small complexes of defined stoichiometry. (*b*) As the cell progresses into mitosis, polo kinase phosphorylates the key scaffolding proteins, thereby inducing their incorporation around the established inner PCM layer. This new addition of protein causes the PCM to expand dramatically. As the mitotic PCM expands, Aurora A kinase phosphorylation promotes the deposition of protein complexes that aid in nucleating and stabilizing MTs (blue lines). The steady-state size of the PCM is determined by the total amount of a limiting component and the rates of phosphorylation and de-phosphorylation reactions. (*c*) Once mitosis is complete, MT-mediated cortical forces rupture the PCM, and protein phosphatases remove polo kinase-derived phosphorylations from the scaffold proteins. These two activities promote rapid disassembly of the mitotic PCM. (Illustration courtesy of Julia Eichhorn.)

The complexity and seemingly disorganized nature of the PCM has made determining its underlying structure and the mechanism of its assembly challenging. As investigations become more sophisticated and more of the complex puzzle is solved, common themes are emerging across the tree of life (figure 3).

Assembly of PCM begins with the deposition of an ordered layer approximately 100 nm thick that encases the centrioles. As the cell cycle advances towards mitosis, polo kinase phosphorylation transforms the cytosolic PCM proteins into assembly-competent building blocks, thus driving their deposition around the already established inner PCM layer. SPD-5, PCNT/D-PLP, Cnn/CDK5RAP2 and Asterless/Cep152 play important scaffolding roles and are presumed to interconnect to form a lattice-like network that establishes the structural foundation of the PCM. Interestingly, based on comparing amino acid sequences across phyla, *C. elegans* lacks PCNT, Cnn and Asterless, proteins that are essential for PCM formation in *Drosophila* and humans. Instead, PCM formation in *C. elegans* depends on the expression of SPD-5, a 135 kDa coiled-coil protein that is not expressed outside of nematodes. Considering that PCNT, Cnn, Asterless and SPD-5 all contain numerous coiled-coil domains, it may be that SPD-5 assumes the function of these proteins in forming the comparatively simplified *C. elegans* centrosome. Furthermore, like PCNT and Cnn, SPD-5 is a key target of polo kinase and its phosphorylation is essential for PCM expansion (J. Woodruff and O. Wueseke 2014, unpublished data). *In vitro* comparison of the structures and biochemical activities of purified PCNT, Cnn, Asterless and SPD-5 will be essential before drawing any concrete connection between them. Nevertheless, we can conclude that PCM expansion is probably driven by the assembly of a few scaffolding proteins. Polo kinase and SPD-2, both of which are conserved proteins, regulate this process. In embryonic systems, the rates and final sizes of centrosomes are determined by limiting amounts of one or more of these proteins.

How PCM disassembles in a timely fashion at the end of mitosis is less clear. Dephosphorylation probably plays a major role in driving the PCM building blocks out of the assembly-competent state and thus dissolving the centromatrix. Additional pathways, including MT-mediated cortical forces could assist this process by rupturing the PCM, thus making the building blocks more accessible to protein phosphatases. We also cannot discount the possibility of protein degradation in controlling the levels of PCM protein, including polo kinase. This topic of research has only just begun, making it difficult to ascribe a specific mechanism to PCM disassembly or even speculate on the conservation of this process.

### (b) Microtubule nucleation in the twenty-first century

The historical role of the PCM has been to nucleate MTs needed for spindle assembly, spindle positioning and intracellular transport. So how does our understanding of PCM structure and dynamics affect our view of MT-nucleation within the PCM? The traditional view proposes that the PCM serves as a binding platform for γ-tubulin-containing complexes that induce MT nucleation. Certainly, γ-tubulin associates with PCNT and SAS-4, and elimination of γ-tubulin reduces MT mass extending from the centrosome [31,32,82]. So far, however, γ-tubulin complexes are poor MT nucleators *in vitro*, and MT asters can still form in the absence of γ-tubulin *in vivo*, indicating that additional mechanisms exist to nucleate MTs (reviewed in [83]). Tumour overexpressed gene (TOG) proteins such as chTOG/ZYG-9 localize to the PCM, bind MTs directly and increase MT polymerization rates and nucleation *in vitro*, making them attractive candidates to regulate MT nucleation within the centrosome. Additionally, it has been shown that SAS-4/Cnn/Asterless/D-PLP-containing (S-CAP) complexes bind to *α*/*β* tubulin dimers [31]. Emerging evidence suggests that these S-CAP complexes form the underlying PCM lattice, meaning that the PCM has an abundance of *α*/*β* tubulin-binding sites. Diffusion of *α*/*β* tubulin dimers into the PCM and subsequent interaction with these binding sites could promote concentration of tubulin far above cytoplasmic background levels and thus favour spontaneous MT nucleation within the PCM. Indeed, *in vivo* fluorescence correlation spectroscopy and *in silico* models suggest that a similar mechanism regulates steady-state concentration of PLK-1 within the PCM [71]. It is possible, then, that the PCM network can concentrate tubulin to the point where MTs spontaneously nucleate. In this model, the role of γ-tubulin could be to cap MTs to provide stability and protect them from depolymerization at the minus ends, while additional MAPs stabilize MTs and drive polymerization of the plus ends.

### (c) The pericentriolar material going forward

As seen for analysis of the PCM structure, novel techniques or approaches play a tremendous role in advancing a field. Emerging techniques such as selective chemical cross-linking (e.g. BioID and S-CROSS) [84,85] and cryo-electron tomography combined with focused-ion-beam milling [86] will certainly yield high-resolution information about the structure and hierarchy of PCM proteins *in situ*. But, the pressing mechanistic questions regarding PCM dynamics and function also require an experimental approach that permits precise control over the molecular machinery therein. *In vitro* reconstitution of a minimal PCM system would grant this kind of accessibility and control. The open questions addressable by such a system are numerous. What are the minimal requirements needed to initiate PCM assembly? How does phosphorylation affect the connectivity and activity of the major scaffold proteins? How does the PCM concentrate *α*/*β* tubulin and drive the nucleation of MTs in the absence of γ-tubulin? Furthermore, structural analysis of PCM assembled from minimal components *in vitro* could also yield atomic-scale resolution of the ‘centromatrix’ and its constituent proteins.

The study of PCM dynamics and structure will certainly clarify how centrosomes serve as MT-organizing centres, trafficking hubs and signalling platforms for the eukaryotic cell. Hopefully, the lessons learned from centrosomes will also shed light on the basic biophysical principles behind the formation of self-assembling macromolecular complexes, including other non-membrane-bound organelles.
